# Alström syndrome: the journey to diagnosis

**DOI:** 10.1186/s13023-024-03509-y

**Published:** 2025-01-06

**Authors:** Akshat Sinha, Kerry Leeson-Beevers, Catherine Lewis, Elizabeth Loughery, Tarekegn Geberhiwot

**Affiliations:** 1 Department of Diabetes, Endocrinology and Metabolism, University Hospitals Birmingham NHS Foundation Trust, Queen Elizabeth Hospital, Birmingham, B15 2TH UK; 2 College of Medicine and Health, University of Birmingham, Vincent Drive, Edgbaston, Birmingham, B15 2TT UK; 3 Institute of Metabolism and System Research, University of Birmingham, Birmingham, UK; 4 Alström Syndrome UK, 4 St Kitts Close, Torquay, Devon, TQ2 7GD UK

**Keywords:** Alström syndrome, Cardiomyopathy, Retinopathy, Obesity, Rare disease, Qualitative, Diagnostic pathway, Referral protocol

## Abstract

**Background:**

Alström syndrome (AS) is a recessively inherited genetic condition which is ultra-rare and extremely complex. Symptoms include retinal dystrophy, nystagmus, photophobia, hearing loss, obesity, insulin resistance, diabetes and cardiomyopathy. The condition is progressive, but it is important to note that not all the complications associated with AS occur in everyone affected. Symptoms can also present at different stages, making diagnosis difficult. There are currently 88 people diagnosed with AS in the UK.

**Objectives:**

The aim of this report is to raise awareness of the key symptoms of AS, in order to promote a faster and more effective diagnosis. This involves identification of individual or a combination of ‘red flag’ symptoms. Overall the findings should improve the patient experience, and their long-term health outcomes.

**Methods:**

Between August-October 2022 we conducted research into a sample of patients from the ASUK database. The process involved a combination of interviews with families, social care and education reviews. Interviews were semi-structured using open questions and a patient-centred approach.

**Results:**

Seventeen newly diagnosed patients were included in our sample. Only 24% of patients were diagnosed within one year following the onset of AS symptoms. Patients with visual impairment and cardiomyopathy were diagnosed much more quickly, either in infancy or early childhood. 41% of our research participants waited over 5 years for a diagnosis. Insufficient research and treatment advances can further impede the diagnostic process and limit access to therapies or clinical trials, ultimately impacting patient outcomes.

**Conclusion:**

While we welcome these developments, our findings, and the evidence we have gathered in this report suggests that more needs to be done to improve the experiences of people receiving a diagnosis of AS. Obesity rapidly developing in infancy should be flagged as a key symptom to be aware of where AS is a possible diagnosis. Visual impairment (88%) in combination with cardiomyopathy (59%) is a frequent first presentation for patients with AS. Most patients (7/17) are diagnosed many years after symptom onset (5–20 years).

## Introduction

Alström syndrome (AS) is an ultra-rare genetic condition that is inherited in an autosomal recessive pattern [1]. The pathophysiology of this disease is extremely complex and involves the *ALMS1* gene. Due to the ubiquitous expression of the *ALMS1* protein in various tissues, multiple body systems are affected. Ocular manifestations of the disease include rod-cone dystrophy, photophobia and nystagmus. Sensorineural hearing loss is also common in Alström patients. Metabolic complications associated with this syndrome include: childhood obesity, hepatic steatosis, hypertriglyceridemia, and insulin resistance which in turn leads to early-onset diabetes [2, 3].

Disorders of the endocrine system have also been studied, revealing decreased production of reproductive, thyroid and growth hormones. Furthermore, the cardiac system is also implicated with the development of infantile cardiomyopathy, which can regress and return once again as a dilated cardiomyopathy in adulthood [4]. Approximately 40% of neonates experience heart failure between the ages of 4 weeks to 3 months [5]. In addition kidney disease often progresses as patients age, until dialysis or transplantation are needed.

Importantly, the condition is progressive; however, not all related complications occur in every AS patient. Symptoms can present at different stages of life, making diagnosis difficult in many cases. The prevalence of AS is thought to be ~ 1 in 1,000,000 [6]. Currently, 88 people have been diagnosed with AS in the UK (March 2023). Delayed diagnosis can have devastating and life-threatening consequences. The impacts of this on patients, carers and family members should not be underestimated.

Accurate and timely diagnosis is essential coupled with access to support networks, medical expertise and information distribution. Appropriate treatment and management of symptoms can improve health outcomes and lead to a better quality of life. Within this report, numerous key observations supported by specialist clinicians have been identified. In order to better understand the reasons for delayed diagnosis, we undertook several interviews with families affected by AS, and mapped their journeys to diagnosis.

Our aims included investigating the timeline and routes to diagnosis. Alongside this, aspects such as health equity, care pathways and disease awareness among care providers will be covered. Observing overall presentation can elucidate key symptoms. Recognition of key ‘red flag’ features or symptom clusters e.g. a combination of visual impairment and obesity will aid early genetic testing. An end goal of improved long-term outcomes and better patient experience is strived for.

## Methods

Between August-October 2022, we conducted research on a sample of patients from the Alström UK (ASUK) database. ASUK is a charitable organisation funded by NHS England. Patients included were diagnosed between 1st April 2020- 1st December 2022. Those already diagnosed and registered on the ASUK database were excluded from this sample, leaving 17 newly diagnosed patients. They were from various locations in the UK and from different demographic sources. Techniques involved qualitative methods such as interviews with families. Demographic data was analysed from hospital, social care and education reviews. Interviews were conducted over telephone by project co-ordinator EL, and semi-structured utilising open-ended questions and a patient-centred approach to explore experiences. The questions focused on patients’ journeys and covered multiple aspects of AS care.

These included the journey to diagnosis, coordination of care, awareness and access to treatment, services, information, and support. Examples of these questions were: “Tell us about your journey to diagnosis with AS?” and “ What individuals were involved in confirming the diagnosis?”. Answers were logged in a pre-designed data extraction sheet using Microsoft Excel. Seventeen patients were diagnosed during this period and were representative of the diversity of patients diagnosed on the ASUK database, particularly in regard to ethnicity (Table 1). The age range in this sample was 7 months to 20 years.

## Results

**Fig. 1:**
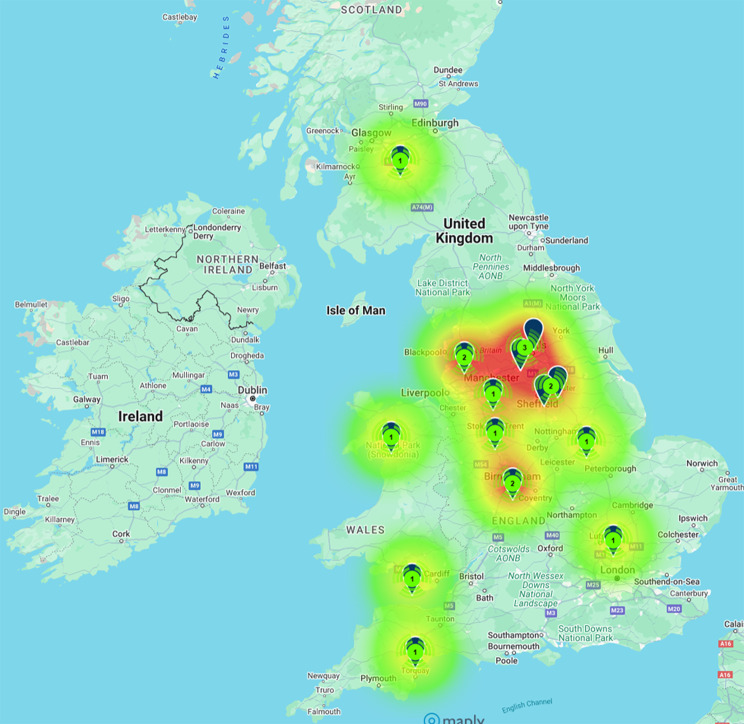
Sample by Location. Heatmap showing areas of UK that Alström syndrome patients are living in

Of the seventeen patients included, most were of White British descent (8/17 [47%]). Other ethnicities comprised of Pakistani (3/17 [17%]), Iraqi (1/17 [6%]), Romanian (1/17 [6%]), Syrian (1/17 [6%]) and Mixed (1/17 [6%]). Two patients did not disclose their ethnicity. All patients’ current ages ranged from 7 months to 20 years. The geographical locations of the samples varied from all parts of the UK, with many patients residing in the northern parts of the UK. Patients were also seen from Scotland and Wales (Fig. 1).

**Table 1:** Demographics of participants

Patient Ethnicity	Number	Proportion
White-British	8	47%
Asian-Pakistani	3	17%
*Asian-Iraqi*	*1*	*6%*
*Asian-Syrian*	*1*	*6%*
*Romanian*	*1*	*6%*
*Mixed*	*1*	*6%*
*Not Disclosed*	*2*	*12%*

### Timeline of diagnosis

**Fig. 2:**
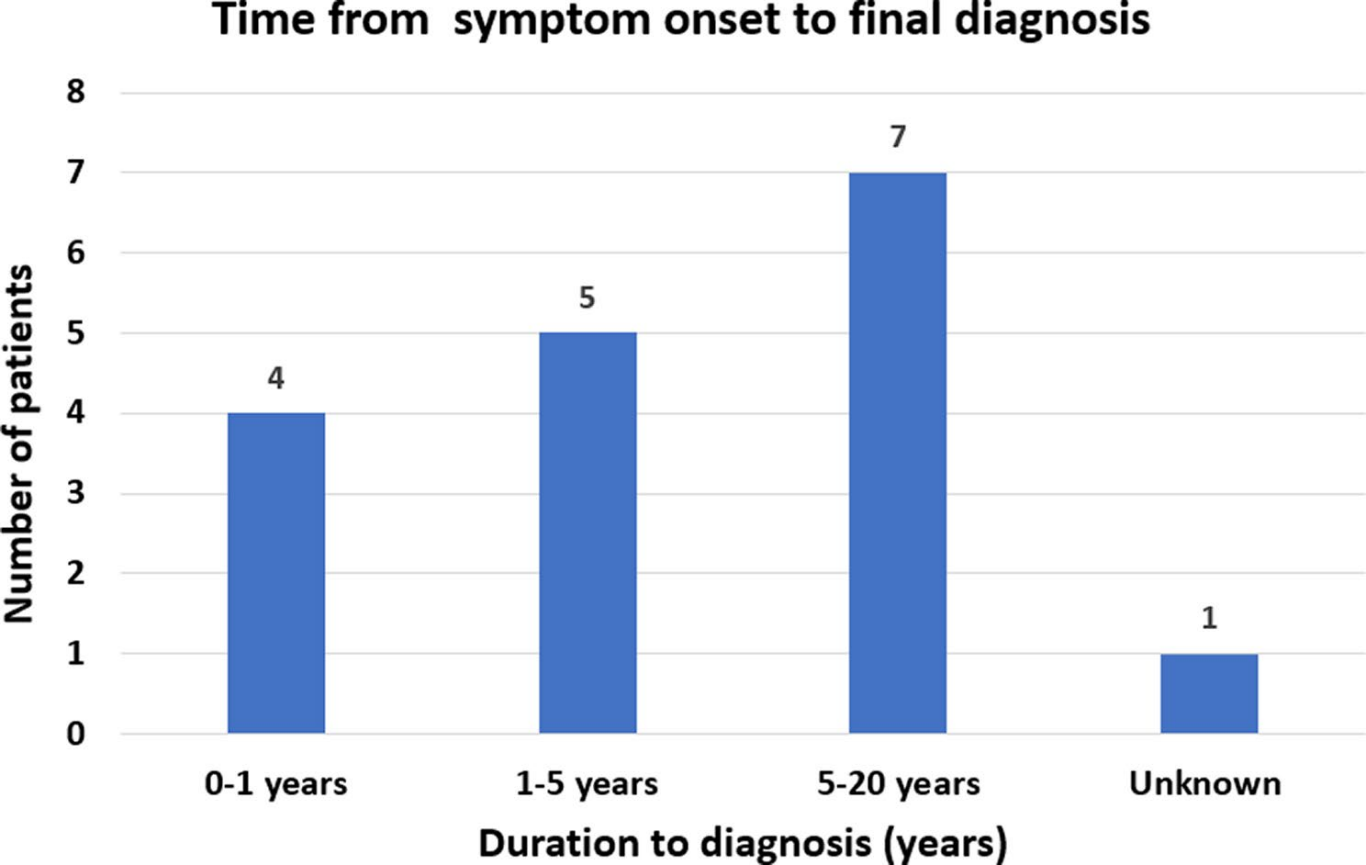
Time from symptom onset to diagnosis. Time spanning from first symptom to Alström syndrome diagnosis

Subsequently, we interpreted waiting time in relation to the time from first symptom onset to diagnosis. In most cases, retinopathy starts in infancy. 41% (7/17) of patients did not receive a diagnosis until 5–20+ years after the initial symptom presentation. 29% (5/17) were diagnosed within 1–5 years, while only 24% (4/17) received a diagnosis in the first year following symptom onset (Fig. 2). For one patient, we were unable to obtain a timeline.

### Route to diagnosis and symptom onset

**Fig. 3:**
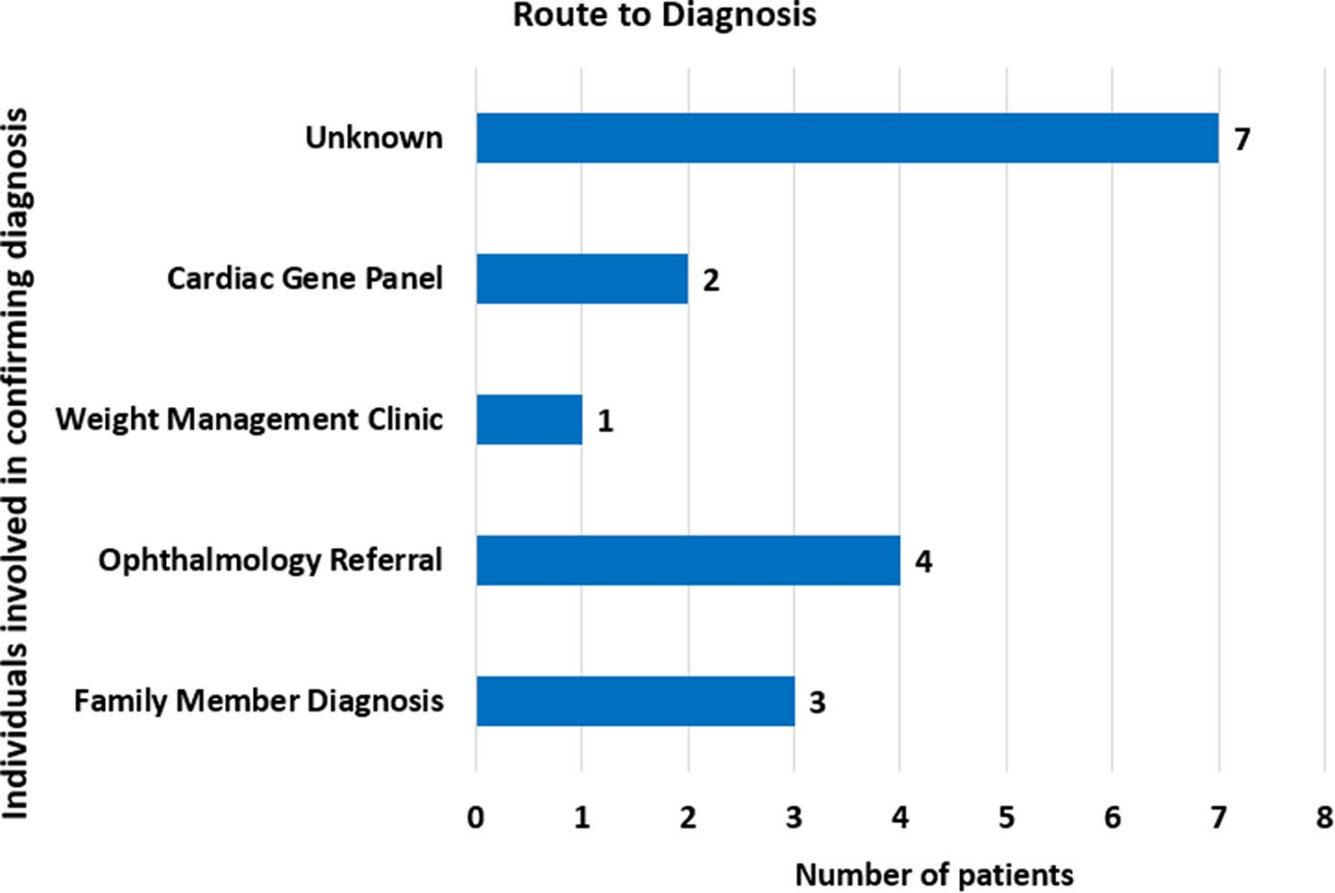
Route to diagnosis. Route demonstrating the healthcare professional or specialist where the diagnosis was confirmed

As well as this establishing key determinants of successful AS diagnosis as part of the patient pathway was observed. The most common route of diagnosis was through ophthalmology referral (Fig. 3). Other individuals involved were GPs, paediatricians, clinicians specialising in weight management, and cardiac geneticists. Cardiac gene panels provided 12% (2/17) of the patients with a diagnosis following the onset of cardiovascular symptoms in early life (<1 year).

**Fig. 4:**
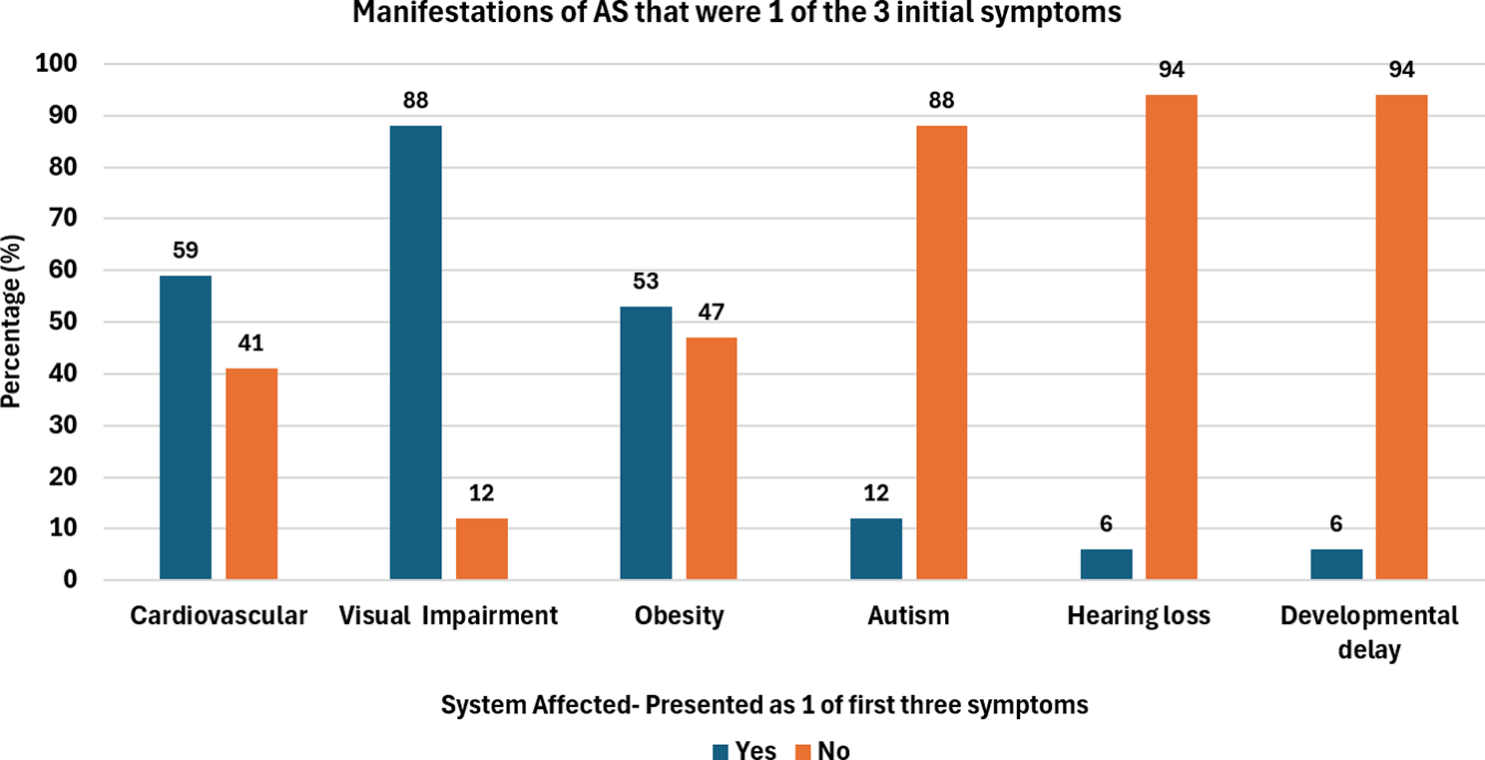
First three initial Alstrom syndrome symptoms. Most common initial symptom that was one of the first three symptoms

In terms of symptoms ***obesity***,*** visual impairment and cardiovascular disease*** were the most commonly reported (Fig. 4). A combination of early-onset cardiomyopathy and visual impairment appeared to lead to a quicker diagnosis, compared with patients in whom these symptoms were absent or had a late onset in our cohort.

### Cardiovascular disease

Dilated cardiomyopathy is one of the most common first AS symptoms with over half (10/17 [58%]) presenting with cardiovascular disease as one of the first three symptoms. The onset of cardiomyopathy can vary from infancy to late childhood and adulthood [7]. Those with early symptoms are typically diagnosed earlier.


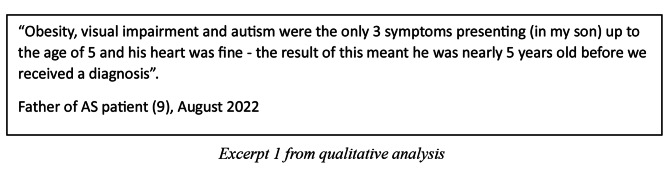


### Obesity

**Fig. 5:**
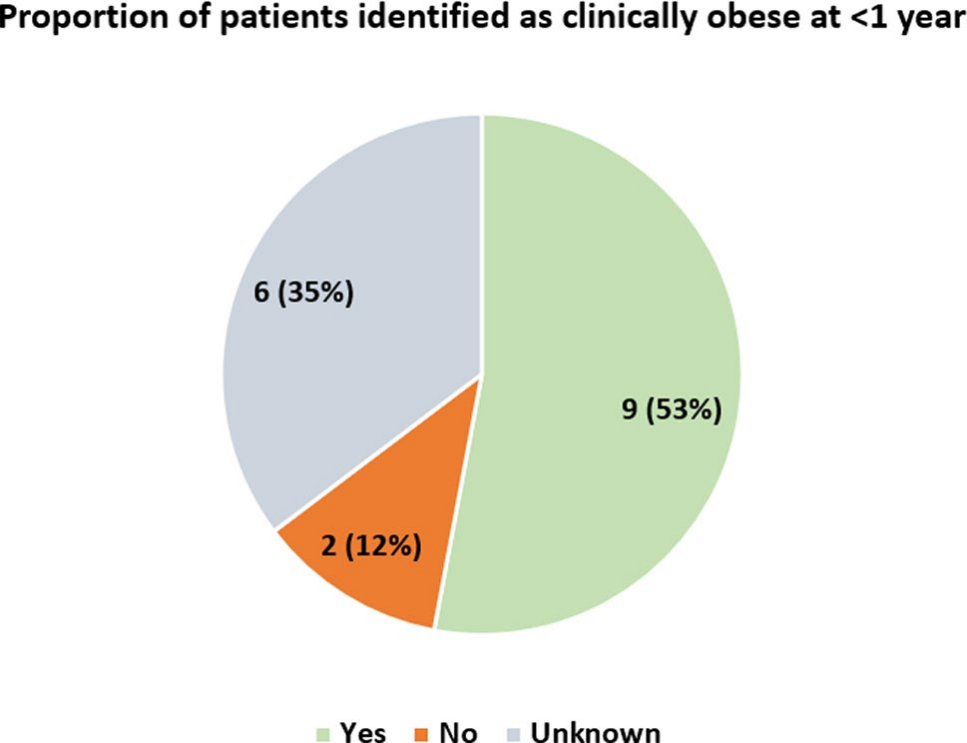
Number identified as clinically obese at < 1 year. Those identified as clinically obese before the age of one in a paediatric evaluation

Obesity is a relatively consistent feature and important early symptom of AS. The majority of patients experienced rapid weight gain in the first year of life. This condition was formally recorded as clinical obesity following paediatric clinic evaluation. More than half of our sample presented with excessive weight gain (Fig. 5). Patient 17 of this study was identified through such a clinic, and with input from ASUK, a direct sibling and another family member were also diagnosed. Access to such clinics proved to be a significant factor in confirming AS in this case.

### Visual impairment

Visual impairment was one of the first three symptoms reported in 88% (15/17) of patients included. Again more than half displayed rod-cone dystrophy before the age of 1, resulting in photophobia and nystagmus [8]. Nevertheless, if this was an isolated symptom patients waited longer for diagnosis in comparison to a cluster of symptoms mentioned above.

## Genetic tests

Of all the patients, 3/17 (18%) were successfully identified through genetic panel testing before the age of 1. Unfortunately, in some cases while testing provides closure, not all experiences were positive. Letters often use complex terminology and are somewhat lacking in information about AS. Similarly, little emphasis is placed on signposting to highly specialised services, which leads carers and families needing to seek alternative sources of knowledge to facilitate understanding of the care pathway.

### Establishing a diagnosis

**Fig. 6:**
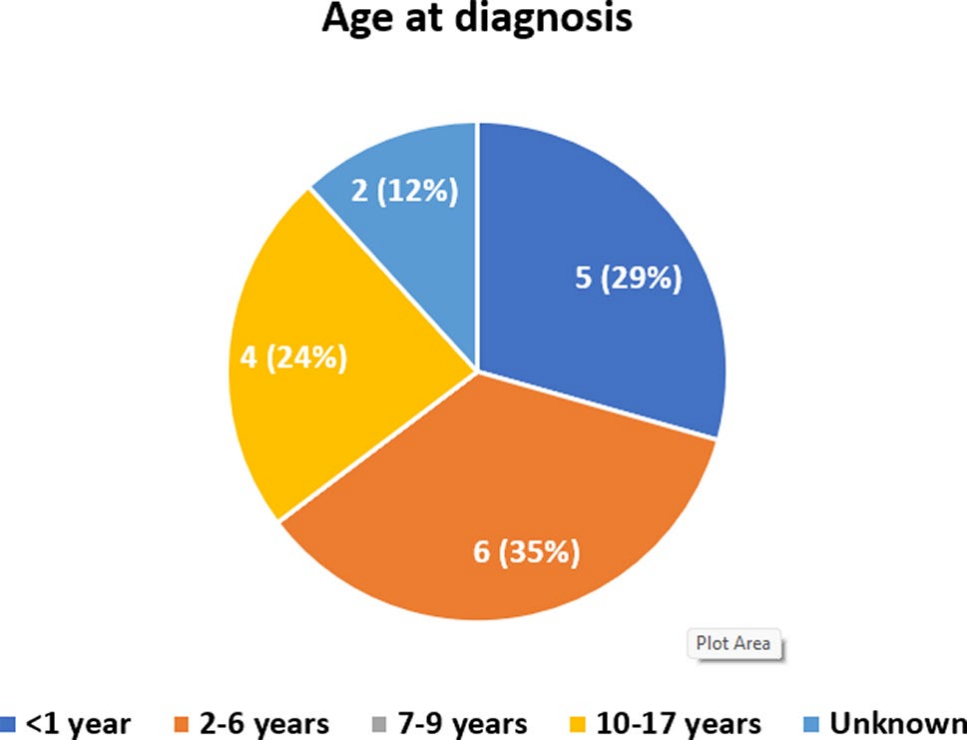
Age when Alstrom syndrome diagnosis confirmed. Final age at time of diagnostic confirmation

Previously we documented the average time to diagnosis, but the age of patients is equally as important. There can be significant delays in accessing this. Only 29% (5/17) of AS cases are diagnosed in the first year of life. Thirty-five percent (6/17) were diagnosed between 2 and 6 years, and 24% (4/17) between the age of 10–17 years (Fig. 6). A total of 2/17 (12%) patients in our study received an incorrect diagnosis of Bardet-Biedl Syndrome and Leber Congenital Amaurosis before further genetic tests confirmed AS. Research from ASUK underlined the fact that families attend numerous GP and hospital appointments before AS is diagnosed. Due to inconsistencies in healthcare practitioners, the lack of continuity of care meant effective holistic review of the patient was not taking place. Patients are frequently diagnosed in institutions far from home.

## Discussion

The average age of diagnosis, reassuringly, is decreasing. In 2022, ASUK data found that the average age of those patients newly referred with AS was seven years. Compared to 2018, this represents a significant reduction from an average of twenty years. Still it is of utmost importance to ascertain the barriers that exist, and find solutions to reduce the age of diagnosis further.

## Barriers to diagnosis

Specifically, a barrier noted is the distinct lack of awareness among healthcare professionals concerning referral to appropriate specialists. In some cases patients, were not referred at all. Providing training on dealing with unusual symptoms and making resources available that describe the referral process may offer a solution. ASUK is regularly the first point of contact; but this is dependent on independent research and families’ confidence to reach out.


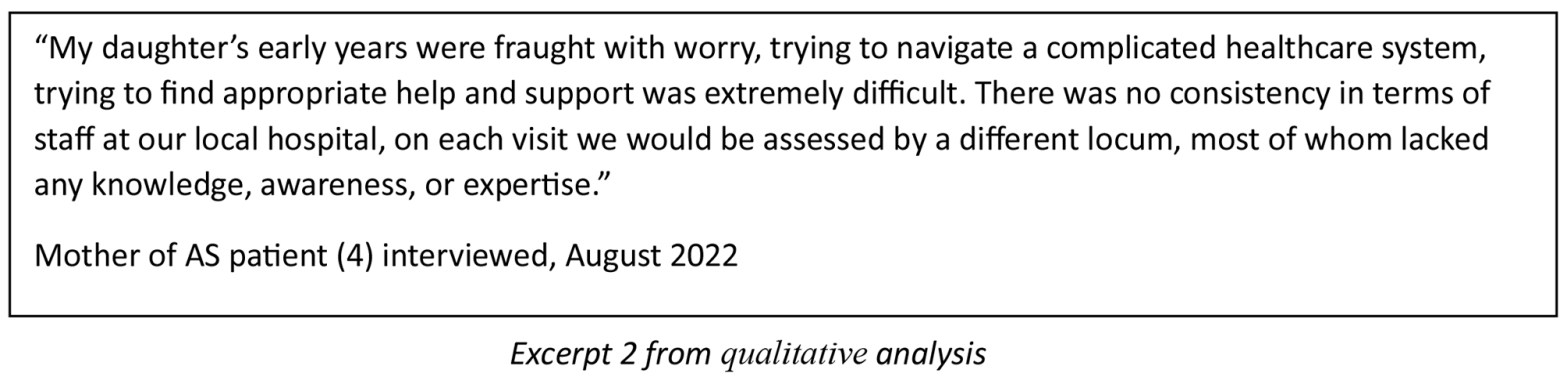


Health inequalities are growing in the UK. A report published by the NHS Race and Health Observatory gathered evidence to illustrate ethnic inequalities in healthcare [10]. The most diverse and marginalised communities tend to be at the greatest disadvantage in accessing healthcare. Its findings have shown that people who are from diverse and marginalised communities and affected by a rare or genetic condition, often experience a huge disadvantage in areas such as genetic testing, mental health, and access to healthcare services.

Our research has identified similar findings - we noted a considerable variance in the ‘patient experience’ in terms of access to quality, effective care, and the speed of diagnosis. This variance linked to factors such as ethnicity, education level, awareness, accessibility, and patient place of residence appears to demonstrate further barriers to a timely diagnosis. Often education plays a role with people from a lower socioeconomic background disproportionately affected.

Being from an isolated, rural location has given patients with AS limited resources, with diagnostic capabilities available restricted to large hospitals. From our sample, *patient 4* was based in a rural and remote area of the UK, where expertise in AS, or rare conditions in general, may be limited. This resulted in a particularly challenging journey to diagnosis. Parents feel they received substandard medical care from their local hospital due to inconsistent practices, and a lack of medical staff with specialist skills to provide effective treatment and appropriate referrals. Only due to the persistence of the parents, was patient 4 referred to a larger hospital to be assessed by appropriate specialists. It was at this hospital that the patient was finally diagnosed.

Stigma surrounding certain cultural practices can strain families’ rapport with healthcare professionals. Certainly, in consanguineous relationships, parents can attach blame to themselves and opt out of genetic testing due to fear of judgement. One family shared their uphill struggle in seeking a diagnosis. Sadly, this family felt they were at risk of being blamed for their child’s condition. The parents were related by blood and acutely aware of the stigma surrounding consanguinity. Due to their lived experience and the perceived lack of cultural competence among medical professionals, parents do not feel that engagement and communication between them and healthcare professionals has been positive. Therefore, they have opted out of having genetic testing (although their child has received a clinical diagnosis). Cultural competence is improving amongst clinicians, and more so than ever they are aware of the diverse practices and worldviews individuals may have.

More recently the COVID-19 pandemic has impacted multiple families. Over half of the families included in this sample reported delays they believed the pandemic had caused in access to treatment, diagnosis and support. Even after the lockdown, many face-to-face consultations were cancelled, compounding feelings of worry, anxiety and isolation. A byproduct being the missed opportunity for diagnosis and counselling of patients in person. The ability to communicate directly with healthcare professionals was diminished.

On the other hand, some positives have come about as a result. The burgeoning area of telemedicine has enabled families to negate long-distance travel, and be seen in a comfortable environment [9]. Acceleration in this area should prevent the troubles associated with getting to appointments due to mobility and vision issues.

## Recommendations

Targeted education and training are essential for raising awareness of AS and other rare conditions, encouraging clinicians to always consider such disorders as part of their differential diagnoses. Continually evaluating and updating current AS clinical guidelines can disseminate information linked to key diagnostic criteria, which should raise suspicion for timely referral and genetic testing. Standardised protocols level out the differences that can contribute to health inequalities. When it comes to genetics, there needs to be a clear structure on what type of tests should be ordered depending on the circumstances. Adopting a multidisciplinary approach improves collaboration, involving various specialists in charge of patient care [11, 12].

Additionally in this digital age, lack of access should no longer be an excuse. The usage of virtual consultations can bridge this gap between symptom onset to diagnosis; especially in areas with scarce access to specialist services. Comprehensive assessments can minimise error and avoid incorrect conclusions [13]. A rapport of trust and openness between families and clinicians can alert doctors to certain concerns and observations, enriching the patient experience. Examining the holistic aspects can ascertain more facets such as the social, mental and physical effects of AS.

What is more is that growing interest can prompt more research in this area to advance treatments, and open access to clinical trials of therapies [14]. A synthesis of the instances where cardiac transplantation has been successful can provide scientific evidence that can build for the future.


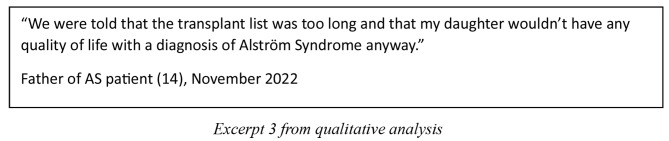


NHS England can endorse charities such as ASUK, to drive clinicians to signpost resources for newly diagnosed patients and families. Mandatory training on cultural competence and humility is a practical solution to ameliorate understanding of patients’ background [15]. The Breaking Down Barriers Experts by Experience Advisory Group provides individuals from diverse backgrounds, to give input when developing services.

Notably the symptoms identified should be added, with extra weight placed on the first key features to present. Liaising with paediatric clinics can expedite the finding of potential AS cases. Ultimately exploring the possibility of AS being included in newborn screening would be the gold-standard for accelerating diagnosis [16]. Lessons learned from the past should be reflected on going forward, to present the best chances for all AS patients. Hopefully, new ideas and concepts can be designed in the coming years revolutionising the outlook on outcomes in ultra-rare conditions.

Limitations of this research stemmed from the retrospective nature of data collection. Often such qualitative projects can be prone to recall bias, which can be more pronounced if diagnosis was particularly delayed. In addition, the sample size of seventeen is small, however AS is an ultra-rare condition. Future projects should focus on more expansive analysis of all UK patients, perhaps including Europe as well for collaborative benefit.

## Conclusion

It is important to learn from patient, parent, and carers’ experiences of diagnosis and to understand how these experiences impact health outcomes and quality of life. Key symptom clusters that often present in AS are obesity, visual impairment and cardiomyopathy. Most commonly, the diagnosis was a result of specialist referral to ophthalmologists. On average only 24% received a diagnosis within one year of symptom onset. Consequently, in our sample the most frequent age at diagnosis was between 2 and 6 years.

Findings in this report highlight where some of the gaps occur, and identifies areas within the health service that require improvement. It also helps us to recognise the areas of good practice and find ways to build on these to improve diagnosis and care pathways for people living with Alström syndrome.

## Data Availability

All data generated or analysed during this study are included in this published article [and its supplementary information files].

## References

[1] Marshall JD, Beck S, Maffei P, Naggert JK. Alström Syndrome. Eur J Hum Genet. 2007;15(12):1193–202. 17940554 10.1038/sj.ejhg.5201933

[2] Paisey RB, Carey CM, Bower L, Marshall J, Taylor P, Maffei P, et al. Hypertriglyceridaemia in Alström’s syndrome: causes and associations in 37 cases. Clin Endocrinol (Oxf). 2004;60(2):228–31. 14725685 10.1111/j.1365-2265.2004.01952.x

[3] Collin GB, Marshall JD, Ikeda A, So WV, Russell-Eggitt I, Maffei P, et al. Mutations in ALMS1 cause obesity, type 2 diabetes and neurosensory degeneration in Alström syndrome. Nat Genet. 2002;31(1):74–8. 11941369 10.1038/ng867

[4] Brofferio A, Sachdev V, Hannoush H, Marshall JD, Naggert JK, Sidenko S, et al. Characteristics of cardiomyopathy in Alström syndrome: prospective single-center data on 38 patients. Mol Genet Metab. 2017;121(4):336–43. 28610912 10.1016/j.ymgme.2017.05.017 PMC5555226

[5] Tahani N, Maffei P, Dollfus H, Paisey R, Valverde D, Milan G, et al. Consensus clinical management guidelines for Alström syndrome. Orphanet J Rare Dis. 2020;15(1):253. 32958032 10.1186/s13023-020-01468-8 PMC7504843

[6] Marshall JD, Maffei P, Beck S, Barrett TG, Paisey R, Naggert JK. Clinical utility gene card for: Alström Syndrome - update 2013. Eur J Hum Genet [Internet]. 2013 Nov [cited 2024 Feb 3];21(11). https://www.ncbi.nlm.nih.gov/pmc/articles/PMC3798853/10.1038/ejhg.2013.61 PMC3798853 23612576

[7] Nerakh G, Ranganath P. Alström Syndrome presenting as isolated dilated cardiomyopathy. Indian J Pediatr. 2019;86(3):296–8. 30484169 10.1007/s12098-018-2807-9

[8] Nasser F, Weisschuh N, Maffei P, Milan G, Heller C, Zrenner E, et al. Ophthalmic features of cone-rod dystrophy caused by pathogenic variants in the ALMS1 gene. Acta Ophthalmol (Copenh). 2018;96(4):e445–54. 10.1111/aos.13612 29193673

[9] Aghdam MRF, Vodovnik A, Hameed RA. Role of Telemedicine in Multidisciplinary Team meetings. J Pathol Inf. 2019;10(1):35. 10.4103/jpi.jpi_20_19 PMC6883478 31799021

[10] Kapadia D, Zhang J, Salway S, Nazroo J, Booth A. Ethnic inequalities in Healthcare: A Rapid Evidence Review.

[11] Van Groenendael S, Giacovazzi L, Davison F, Holtkemper O, Huang Z, Wang Q, et al. High quality, patient centred and coordinated care for Alstrom syndrome: a model of care for an ultra-rare disease. Orphanet J Rare Dis. 2015;10:149. 26603037 10.1186/s13023-015-0366-y PMC4657378

[12] Macken WL, Falabella M, McKittrick C, Pizzamiglio C, Ellmers R, Eggleton K, et al. Specialist multidisciplinary input maximises rare disease diagnoses from whole genome sequencing. Nat Commun. 2022;13(1):6324. 36344503 10.1038/s41467-022-32908-7 PMC9640711

[13] Wen J, Schulman KA. Can Team-Based Care improve patient satisfaction? A systematic review of Randomized controlled trials. PLoS ONE. 2014;9(7):e100603. 25014674 10.1371/journal.pone.0100603 PMC4094385

[14] Baig S, Veeranna V, Bolton S, Edwards N, Tomlinson JW, Manolopoulos K, et al. Treatment with PBI-4050 in patients with Alström syndrome: study protocol for a phase 2, single-Centre, single-arm, open-label trial. BMC Endocr Disord. 2018;18(1):88. 30477455 10.1186/s12902-018-0315-6 PMC6258144

[15] Jongen C, McCalman J, Bainbridge R. Health workforce cultural competency interventions: a systematic scoping review. BMC Health Serv Res. 2018;18:232. 29609614 10.1186/s12913-018-3001-5 PMC5879833

[16] Stark Z, Scott RH. Genomic newborn screening for rare diseases. Nat Rev Genet. 2023;24(11):755–66. 37386126 10.1038/s41576-023-00621-w

